# Animal Models in the Evaluation of the Effectiveness of Phage Therapy for Infections Caused by Gram-Negative Bacteria from the ESKAPE Group and the Reliability of Its Use in Humans

**DOI:** 10.3390/microorganisms9020206

**Published:** 2021-01-20

**Authors:** Martyna Cieślik, Natalia Bagińska, Andrzej Górski, Ewa Jończyk-Matysiak

**Affiliations:** 1 Bacteriophage Laboratory, Ludwik Hirszfeld Institute of Immunology and Experimental Therapy, Polish Academy of Sciences, 53-114 Wroclaw, Poland; martyna.cieslik@hirszfeld.pl (M.C.); natalia.baginska@hirszfeld.pl (N.B.); andrzej.gorski@hirszfeld.pl (A.G.); 2 Phage Therapy Unit, Ludwik Hirszfeld Institute of Immunology and Experimental Therapy, Polish Academy of Sciences, 53-114 Wroclaw, Poland

**Keywords:** animal models, in vivo studies, phage therapy, ESKAPE group, antibiotic resistance

## Abstract

The authors emphasize how extremely important it is to highlight the role played by animal models in an attempt to determine possible phage interactions with the organism into which it was introduced as well as to determine the safety and effectiveness of phage therapy in vivo taking into account the individual conditions of a given organism and its physiology. Animal models in which phages are used make it possible, among other things, to evaluate the effective therapeutic dose and to choose the possible route of phage administration depending on the type of infection developed. These results cannot be applied in detail to the human body, but the knowledge gained from animal experiments is invaluable and very helpful. We would like to highlight how useful animal models may be for the possible effectiveness evaluation of phage therapy in the case of infections caused by gram-negative bacteria from the ESKAPE (*Enterococcus faecium*, *Staphylococcus aureus*, *Klebsiella pneumoniae*, *Acinetobacter baumannii*, *Pseudomonas aeruginosa*, *Enterobacter* species) group of pathogens. In this review, we focus specifically on the data from the last few years.

## 1. The ESKAPE Group of Bacteria as a Great Medical Challenge

The overuse of antibiotics and their inappropriate use have contributed greatly to the increase of the observed phenomenon of antibiotic resistance among bacteria. In its reports, the World Health Organization (WHO) highlights that in the near future, due to the lack of means of eliminating bacteria, even minor infections may turn out to be fatal [1]. Moreover, due to the growing drug resistance of microorganisms, more and more economic problems have arisen, including those related to the increasing costs of patient treatment [2], especially in the case of patients with chronic infections caused by extremely resistant bacteria where even last-resort antibiotics do not appear to be effective. It has been estimated that the total cost of antibiotic resistance is approximately $20 billion in direct healthcare costs in the United States alone each year [3].

One of the most serious challenges in the modern medicine is the fight against nosocomial infections with multidrug-resistant bacteria of the ESKAPE (*Enterococcus faecium*, *Staphylococcus aureus*, *Klebsiella pneumoniae*, *Acinetobacter baumannii*, *Pseudomonas aeruginosa*, *Enterobacter* species) group [4,5,6]. These pathogens are extremely dangerous because of the possibility of the appearance of new and more sophisticated mechanisms of drug resistance. The acronym ESKAPE refers precisely to the “escape” of these bacteria from the action of antibiotics [4]. Infections with these bacteria are especially common in hospitalized and/or immunocompromised patients and in those with intestinal flora dysbiosis [7,8] and pose a serious threat to the life and health of these patients.

Two of those pathogens, *E. faecium* and *S. aureus*, are clinically relevant gram-positive bacteria. The remaining ESKAPE species are gram-negative bacteria with many different antibiotic resistance mechanisms.

Our aim is to focus on gram-negative bacteria from the ESKAPE group. *Klebsiella pneumoniae*, formerly known as Friedländer’s bacillus, which is a facultative encapsulated anaerobic rod-shaped bacterium, is responsible for many life-threatening infections such as pneumonia, sepsis, bacteremia, urinary tract infections, meningitis, and liver abscess [9,10]. The presence of β-lactamases is a significant factor causing drug resistance [6]. Plasmid-encoded extended-spectrum β-lactamase (ESBL) enzymes presented in *K. pneumoniae* strains are responsible for failure in the use of penicillins, generation II–IV cephalosporins, as well as of monobactams. Moreover, detection of the *K. pneumoniae* strains producing carbapenemase (KPC), the enzyme which is responsible for resistance to carbapenem (ertapenem, meropenem, imipenem), is associated with even greater therapeutic challenges, because the KPC mechanism also determines resistance to other non-antibiotic drugs [11]. Furthermore, strains producing NDM (New Delhi metallo-β-lactamase) are becoming more common in various countries around the world [12]. An important bacterium of the *Klebsiella* species that is also distributed from hospitalized patients is *K. oxytoca*, which may be responsible for respiratory tract infections, urinary tract infections, and sepsis [13].

*Acinetobacter baumannii* is a short, strictly aerobic coccobacillus which causes high mortality in hospitalized patients, mainly in intensive care units. The most dangerous contagions caused by *Acinetobacter* are infections of the bloodstream, lungs, urinary tract, surgical wounds (infections of skin and soft tissue), and meningitis [14]. The most important antibiotic resistance mechanisms of *A. baumannii* include the production of β-lactamases, which are enzymes that inactivate β-lactam antibiotics, as well as the presence of efflux pumps actively removing drugs from the inside of the cell and the ability to modify membrane proteins associated with the transport of drug molecules [15,16,17,18]. The most frequently isolated strains from genus *Acinetobacter* are *Acinetobacter baumannii*, but *A. pittii* and *A. nosocomialis* are also clinically significant species [14].

*Pseudomonas aeruginosa* is a facultative encapsulated anaerobic rod-shaped bacterium, produces many characteristic pigments, and is especially ubiquitous in the environment [19,20]. The most dangerous difficult-to-treat infections caused by *P. aeruginosa* include pneumonia [21], sepsis [22], urinary tract infections [23], and, additionally, eye, ear, skin, bone, gastrointestinal, and central nervous system infections [24]. Contagions with this bacterium turn out to be particularly menacing for patients suffering from cystic fibrosis, e.g., due to the continuous inflammatory process in the lungs during this disease [25]. Resistance to antimicrobial agents is conditioned by the presence of efflux pumps, expression of the AmpC cephalosporinase hydrolyzing most β-lactams, and the particularly poor permeability of the bacterial membrane [26]. 

*Enterobacter* spp. are facultative anaerobic rod-shaped bacilli which are responsible for some nosocomial infections, such as bacteremia, sepsis, pneumonia, urinary tract infection (especially as a complication after catheterization), postsurgical peritonitis, meningitis, and endocarditis [27]. *Enterobacter* isolates producing extended-spectrum β-lactamases (ESBL) as well as those with the genes conditioning resistance to carbapenems have been described [28]. The most frequent strains in the hospital environment are *E. cloacae*, *E. aerogenes*, and *E. hormaechei* [27,28]. 

Each of the bacteria discussed in the review has the ability to produce a biofilm, which is a significant pathogenicity factor and also promotes antibiotic-resistant bacteria.

## 2. Phage Therapy as Alternative Treatment of Infections Caused by Gram-Negative Bacteria from the ESKAPE Group

In response to the increasing drama of antibiotic resistance, phage therapy has flourished in the past years, and an increased number of articles reporting success have been published. However, no successful clinical trial carried out in accord with the current evidence-based medicine standards has formally proved the clinical efficacy of phage therapy. Therefore, critical analysis of the data derived from experimental studies in animals is important to plan a trial with greater chances of success [29]. 

In addition to complete phage particles, enzymes derived from bacteriophage genomes, such as lysins or depolymerases, can be effective tools for fighting bacteria because of their hydrolytic activity in peptidoglycan degradation. Endolysins are promising potential therapeutic agents to research because of the lack of observation of acquisition resistance to them in bacteria [30]. Another important factor for the effective antimicrobial activity of phages is the expression of depolymerases, i.e., the enzymes responsible for cleaving polysaccharides from the bacterial capsule, such as lipopolysaccharides (LPS) or exopolysaccharides (EPS) [31]. This phenomenon may be of great importance in combating gram-negative bacteria in particular, because LPS constitute an important characteristic component of their outer membrane.

Bacterial endotoxins, e.g., the lipopolysaccharides contained in phage lysates, may constitute a limitation of phage therapy. Lipid A, which is part of the gram-negative lipopolysaccharide (LPS), is responsible for synthesis of different mediators of inflammation, which is expressed by activating macrophages and monocytes that release prostaglandins, interleukins (IL-1 and IL-6), tumor necrosis factor (TNF), platelet-derived growth factor (PDGF), free radicals, and colony-stimulating factors (CSF). Endotoxin-induced overproduction of inflammatory mediators and coagulation factors may lead to septic shock, blood vessel damage, intravascular coagulation, and different organ failure [32,33,34,35]. According to the European Pharmacopoeia, the permissible dose of endotoxins in the preparation is 5.0 IU per kilogram of body mass per hour in intravenous administration [36]. However, new and increasingly effective methods of purifying lysates from endotoxins, such as ultracentrifugation, ultrafiltration, precipitation with polyethylene glycol, octanol extraction, anion-exchange chromatography, or endotoxin removal columns have been developed [37,38,39], making even the intravenous administration of phage preparations safe [40]. Interestingly, the endotoxins may also have positive effects. These toxins have been used to non-specifically improve the immune defense [41]. 

## 3. Usefulness of Animal Models and their Limitations

In the case of phage therapy, it has been emphasized that the timely supply of the product for targeted personalized therapy requires the processes leading to their production and application to be accelerated [42]. In particular, due to the constantly increasing resistance of bacteria to drugs and the emerging problem of phage resistance [43], it is recommended to adapt the therapeutic phages to each case of infection for every single patient [42]. Nevertheless, in vivo studies with the use of laboratory animals are an extremely important element in the studies on the evaluation of the safety and effectiveness of new active molecules. These studies may provide the critical information necessary to evaluate the safety and efficiency of a specific therapy in humans. Properly constructed animal models, including both vertebrates and invertebrates, provide a broader view of the mechanism of phage therapy on a living organism, give information on the impact on the immune system (possible interactions with immune system components, e.g., phagocytes), gut microbiota, infected tissue, and allow estimation of the scale of safety, tolerability, and observation of the possible side effects of the preparation used [44,45,46]. In vitro studies will never fully provide valuable information on drug metabolism, tissue distribution, and bioavailability. For example, it is also impossible to reproduce biofilm conditions in vitro [47]. In order to learn more about new phage preparation, preclinical studies are carried out with laboratory animals. 

*Galleria mellonella* (greater wax moth, honeycomb moth) larvae turn out to be an extremely reliable model of infection with various bacteria. This animal model is a very useful tool during the preclinical testing of novel drugs. *G. mellonella* have an immune response that consists of two parts: humoral and cellular responses [48]. Some similarity of the innate immune response of *G. mellonella* to the immune system of mammals, such as some opsonins (for example, apolipophorin III) or NADPH oxidase-dependent killing of pathogens, makes it a particularly interesting and frequently chosen model [49,50]. Moreover, larvae are undemanding when it comes to nutrition and conditions of their maintenance. Of special importance is that they survive at 37 °C, which is a necessary temperature to culture the majority of human pathogens [51] and corresponds to human temperature. The process of melanization, consisting of the deposition of melanin in the tissues, is compared to the formation of an abscess at the side of an infection in mammals [50]; moreover, it is an important determinant of the effectiveness or ineffectiveness of treatment in the tested agents [52,53,54]. Another invertebrate described in the publications is *Drosophila melanogaster* (fruit fly), which may be useful in testing the safety and/or toxicity of phage preparations and their pharmacokinetics after oral administration [55] or assessing the therapeutic effects of phages after injection [56]. Overall, invertebrate studies are relatively inexpensive, easy to perform, and quick, yet they provide a lot of important information on the therapeutic and prophylactic effects as well as the safety of phage use [44]. Despite many advantages, invertebrate organisms are very different from mammals, including humans, and have many limitations when it comes to translating therapies to humans. *G. mellonella* larvae are treated with both bacteria and phages mainly into the last proleg (examples of which are given below), and therefore the testing of different routes of phage administration is very limited.

Many more benefits can be derived from testing the new therapeutic molecules in vertebrates. Studies in murine models are particularly often described, including in the case of bacterial infections and phage therapy used against them. Many different routes of administration of bacteriophages or their enzymes can be used in mice, for example, intranasally or intratracheally in the case of pneumonia [54,57,58,59,60], intraperitoneally or intramuscularly in the case of sepsis [55,61,62,63], intraperitoneally or/and topically in the case of wound infection [59,64], by injection into subcutaneous pockets in the case of a wound infection sustained during catheterization [65], and topically to the corneal surface in the cases of eye infections [66,67]. The advantages and disadvantages of different routes of phages or phage enzyme administration are presented in Table 1. In vivo studies in mice have also been successfully carried out on the oral administration of liposome-encapsulated phages, which prolonged their persistence in the gastrointestinal tract [68]. The importance of research on the different routes of administration is underlined due to the possible resulting different phage pharmacokinetics, which, as a consequence, may result in more or less effective action [47,69]. In the case of an attempt to translate these studies into human medicine, there are also some limitations related to different conditions in the digestive tract of various animals, including humans, e.g., significantly different pH values of gastric juice [70]. Preclinical animal studies also evaluate the effectiveness of single phages compared to phage cocktails, and more advantages of using phage mix are demonstrated, for example, a wider range of bacterial hosts that the phages can combat [47,71]. It is also possible to compare a single phage dose with multiple doses for a particular infection, and their therapeutic effects may be due to the titer of phages in any doses, phage adsorption rate, or elimination rate [71]. 

In animal models, it is easier to induce acute bacterial infection, while patients receiving phage therapy usually suffer from chronic infections [72]. Furthermore, in the experimental research, bacteriophage preparations have been usually applied a short time after infection (generally after tens of minutes or several hours), examples of which are given below, whereas in the case of patient infection, the period of time between the onset of infection and the start of phage therapy is much longer. The right time assessment of the timepoint at which the phages will be delivered after the development of infection is also important, because a delay in phage application may result in a lack of efficacy of the therapy [73].

Certain diseases cannot be fully reproduced in animal models, an example of which is cystic fibrosis, which has many links to *P. aeruginosa* pneumonia. Otherwise, each animal used in preclinical studies of new therapeutic agents will not fully reproduce the human organism because of their metabolism, gene expression, and organ function [74]. There are reports in the literature on the use of non-human primates in experimental studies that most closely resemble humans, e.g., macaques to study the pathogenesis of periodontal disease [75]. The ethical reports highlight many of the related problems, including the stressful conditions during the transport of monkeys from their natural environment, and propose replacement variants, such as specially designed cells expressing human proteins that are implanted into smaller animals, such as rodents [76]. Despite this, valuable knowledge on many diseases that threaten humans has been gained through research with macaques, including studies of the immune system during HIV infection/AIDS progression [77,78], and in conjunction with HIV vaccine research [79], as well as through learning the details of the immune response in tuberculosis [80]. Another major problem faced in planning research on vertebrate animals is the inevitability of obtaining approval from an ethics committee to work with animals. Application for consent should provide a specific committee with a detailed course of the planned work and each procedure, as well as the degree of invasiveness of the research. In particular, the level of achievable benefits for human medicine that can be achieved through the potential harm to animals is assessed [81]. 

Below are examples and a discussion of recent studies using animal models and phage therapy for infections with gram-negative bacteria from the ESKAPE group.

## 4. Animal Models of *Klebsiella pneumoniae* Infection

Thiry et al. characterized three novel lytic bacteriophages against opportunistic multidrug-resistant or hypervirulent *Klebsiella pneumoniae* isolates: vB_KpnP_KL106-ULIP47, vB_KpnP_KL106-ULIP54, and vB_KpnP_KL1-ULIP33 [82]. *Galleria mellonella* larvae infected with *K. pneumoniae* turned out to be a useful tool in assessing the effectiveness of phages, both prophylactic and therapeutic. The larvae were injected with the bacterial suspension to the last proleg, while the phage inoculation at MOI (multiplicity of infection) = 10 was introduced 1 h later on the other side of the same proleg. Both the group of larvae that received phages after infection and the group that received prophylactic phage therapy showed a significant reduction of mortality when compared to the untreated group. The safety of phage therapy was proven by demonstrating no decrease in the survival rate in the group of larvae receiving only phages.

In another study, Wintachai et al. presented the characterization and estimation of therapeutic effectiveness of the phage KP1801, which was isolated from treated hospital wastewater [52]. Interestingly, the described bacteriophage was lytic and active against multidrug-resistant *Klebsiella pneumoniae* strains producing ESBL (extended-spectrum β-lactamase) enzymes. *G. mellonella* larvae infected with bacteria at a dose corresponding to the LD50 index were used to evaluate the efficacy of the therapy in vivo. As expected, half of the untreated larvae died after five days and marked melanization was observed. It turns out that a single dose of phage preparation administered both as a treatment 2 h after bacteria inoculation and prophylactically 2 h before infection visibly reduced larvae mortality five days after inoculation. Importantly, a clear protective effect of phages was demonstrated, based on an approximate 93–100% survival rate in the larvae that received bacteriophages prior to bacterial infection, while post-infection treatment resulted in the survival of 73–100% of the larvae, in direct proportion to the MOI. Moreover, in both cases, a statistically significant reduction of bacterial load in the larvae and an increase in the number of phages demonstrating their successful replication have been reported.

In a study done by Manohar et al., the potential therapeutic use of the phage KPP235 against *Klebsiella pneumoniae* has been proven using *G. mellonella* larvae [83]. In this research, a single phage dose was enough to completely reduce mortality of the infected larvae over the 96-h observation period, while without treatment, the mortality rate was 100% 48 h post-infection. However, to maintain good larvae mobility, two doses (the second dose was administered 6 h after the first) were required. In addition, two doses of phages were enough to decrease the bacterial count from dead larvae, and after three doses, bacteria were not present. Interestingly, the multiple bacterial infections (*K. pneumoniae*, *E. coli*, and *E. cloacae*) model was applied to assess the effectiveness of a phage cocktail containing three phages against these bacteria. It turned out that the administration of the phage cocktail four times led to a significant improvement in the condition of the larvae.

A murine model of pneumonia caused by virulent *Klebsiella pneumoniae* was used to evaluate the phagotherapy by Anand et al. [57]. A novel lytic phage VTCCBPA43 was isolated from River Ganga. Their therapeutic ability was estimated using BALB/c mice infected by intranasal administration of *K. pneumoniae* suspension, which caused bronchoalveolar pneumonia. In mice that were intranasally administered phages 2 h after infection, a significant reduction of bacterial counts in the lung tissue compared with the untreated group was observed. Moreover, in the histopathological examination of lung sections, marked reduction of neutrophil and lymphocyte infiltration after phage treatment was noted. Furthermore, small areas of necrosis were present in these mice, whereas in the mice treated with bacteria, only the lung lesions were much more severe. No deleterious effects from the use of only phages were shown.

## 5. Animal Models of *Acinetobacter baumannii* Infection

Grygorcewicz et al. described a new bacteriophage vB_AbaP_AGC01 isolated from fishpond water samples and capable of infecting 93 out of 185 *A. baumannii* strains (approximately 50%) which did not infect other bacteria, such as *Klebsiella* spp., *Pseudomonas* spp., *Enterobacter* spp., or *E. coli* [53]. For in vivo studies, *Galleria mellonella* larvae were used, infected by injection of 4 × 10^6^ CFU of the *A. baumannii* suspension behind the last proleg into the hemolymph. The larvae were administered the phage suspension at MOI = 1, MOI = 10, or MOI = 50 on the opposite side of the proleg 20 min after the infection. During the 120-h observation period, a significant reduction of larval mortality was observed. Furthermore, even more interesting results were obtained by administering a combination of phages and antibiotics, especially meropenem or ciprofloxacin. The survival rate of larvae increased most significantly (from 35% to 77%) after combined meropenem and phage (MOI = 10) treatment.

An interesting study by Jeon et al. demonstrated that both *Galleria mellonella* larvae and mouse models are suitable to assess the effectiveness of phage therapy against *A. baumannii* [54]. Phage Βϕ-R2096 isolated from hospital sewage had lytic properties against 16 of the 20 carbapenem-resistant and one of the three carbapenem- and colistin-resistant strains of *A. baumannii*. The examined larvae were divided into different groups. In one of them 30 min after injection of the bacterial suspension in the last proleg, larvae were treated by injection of bacteriophages (1 × 10^10^ PFU/mL, MOI = 100 or MOI = 10) into a different proleg (PFU—plaque-forming unit). After incubation, significant enhancement of the larval survival rate in comparison with the bacteria-only treatment group was observed. Moreover, only slight tissue damage was noted after a single dose of the phage, while in untreated larvae, the damage was much more pronounced. The administration of only the phage did not cause any pathological changes in the tissue. The second authoritative tool used was a mouse model of acute pneumonia. Female C57BL/6 mice were intraperitoneally administered a suspension of bacteria and then, 30 min after infection, treated with bacteriophages (1 × 10^10^ PFU/mL) by intranasal administration. The use of phages resulted in significantly higher MOI-dependent survival rates of mice (100% at MOI = 10, 60% at MOI = 1, 30% at MOI = 0.1) compared to the positive (infected but not treated) control group. Furthermore, only mild damage to the alveolar wall was observed in the phage treatment group, which was clearly less marked than the lesions in the untreated group. It should also be emphasized that the levels of proinflammatory cytokines, such as TNF-α and IL-6, in the lung tissue were significantly reduced following post-infection phage administration.

Wang et al. described a new phage vB_AbaM_IME285 and its depolymerase Dp49 isolated from untreated hospital sewage [86]. BALB/c mice administered intraperitoneally with *A. baumannii* suspensions (Ab387 strain or Ab220 strain) were used in the experiment. The most important effects of IME285 phage therapy 30 min post-infection are the achievement of 100% survival rates after 96 h of observation (both bacterial strains), as well as a significant reduction in the bacterial load in the murine lungs, spleen, and liver (especially in the case of Ab387 infection). Depolymerase Dp49 treatment contributed to the survival of all mice infected with both Ab387 and Ab220 strains and, similar to the abovementioned data, a decrease in the bacterial count of different organs (especially in the case of Ab220 infection).

A mouse model of systemic infection caused by *A. baumannii* and the applied phage therapy was described by Jiang et al. [61]. Immediately after intraperitoneal administration of bacterial suspension, mice were injected with the first dose of the bacteriophage suspension (5.0 × 10^8^ PFU) by the same route. The second dose was administered 12 h later and the monitoring period was 7 days. The survival of treated mice increased, but this result was not statistically significant.

Very interesting research on the use of phage cocktails in dorsal wound infections in BALB/c mice was presented by Rouse et al. [64]. The studies concerned the wound infection caused by *A. baumannii* and aimed to combat it with a phage cocktail containing five lytic bacteriophages (AbArmy φ1, AbNavy φ1, AbNavy φ2, AbNavy φ3, AbNavy φ4). Healthy mice were treated with PBS (negative control) or phages (to check the preventive effect) by intraperitoneal administration, and mice infected with bacteria were treated with PBS (negative control) or phages (to check the therapeutic effect) both by local application under the dressing and injected into the peritoneal cavity. The role of the immune system in response to the prophylactic use of phages was underlined. In general, administration of the phage cocktail reduced the secretion of cytokines and chemokines, such as pro-inflammatory IL-12, IL-13, CCL5, and hemopoietic G-CSF (granulocyte colony-stimulating factor), by immunological cells. Despite this fact, the count of the immunocompetent cells like lymphocytes, monocytes, or neutrophils in the serum did not change significantly. Likewise, a marked change in the number of macrophages, T cells, B cells, and dendritic cells in the liver, spleen, and lymph nodes has not been noted. Prophylactic use of phages influenced induction of the production of IgG2a and IgG2b antibodies by B lymphocytes. The presence of antibodies against bacteriophages was also obtained, which may be related to their neutralization, but does not necessarily indicate the ineffectiveness of the phage therapy [87]. However, application of phages before infection did not significantly reduce the bacterial burden. Post-infection treatment reduced the size of the wound and allowed complete healing within 17 days [64]. The humoral immune response to phages in animals subjected to phage therapy was discussed by Gembara and Dąbrowska [88]. Therapeutic efficacy may be reduced by preexisting natural antibodies following the first phage administration and the repeated administration of phages, especially using the parenteral route.

A mouse model (CD1 Swiss mice) of bacteremia caused by *A. baumannii* was devised by Leshkasheli et al. to evaluate the effectiveness of a new phage therapy [62]. Two novel bacteriophages, vB_AbaM_3054 and vB_AbaM_3090, were obtained from raw sewage water samples. The bacterial suspension was injected intraperitoneally, which resulted in systemic inflammation. The bacteriophage solutions (at MOI = 100), each individually or together, were administered to the mice via the same route 2 h later. After a one-week observation period, high survival rates of mice (in the range of 80–100%) were noted, whereas in the comparison group treated with imipenem, only 17% of the animals survived. This means that both monotherapy and the combination of two phages were more effective in treating sepsis than the commonly known antibiotic.

Wu et al. investigated the use of phage PD-6A3, its endolysin, and a cocktail of 14 phages in the fight against systemic infection caused by *A. baumannii* in BALB/c mice [63]. After a seven-day observation period, the highest survival rates (70%) were found in the mice treated with endolysin and with the combination of endolysin and the PD-6A3 phage. Moreover, in these two groups, the most significant reduction in white blood cell (WBC) count was noted as compared to the untreated mice, which indicates a more effective action of the phage-derived endolysin than of phages. All therapeutic agents were administered into the peritoneal cavity, into which the bacterial suspension had also been introduced.

## 6. Animal Models of *Pseudomonas aeruginosa* Infections

The zebrafish model is also used to ascertain the effects of phage therapy [44,84]. *Pseudomonas aeruginosa* infection is an important complication in cystic fibrosis, which contributes to the high mortality rate of patients [89]. Cafora et al. used a zebrafish embryo model with cystic fibrosis associated with the *P. aeruginosa* infection [84]. The animals were given two doses of a four-phage cocktail into the yolk sac: the first dose 30 min and the second dose 7 h after infection. A significant increase in embryo survival rate was noted for both phage administration times, which gives hope for the application of such a method of therapy in patients suffering from cystic fibrosis complicated by infection. Further, the anti-inflammatory effects of phages were also demonstrated when administered to uninfected embryos expressed by a reduced level of pro-inflammatory cytokines (TNF-α and IL-1β), the concentrations of which are higher in cystic fibrosis.

Jeon and Yong described two bacteriophages, Bϕ-R656 and Bϕ-R1836, against *Pseudomonas aeruginosa* infection in both *Galleria mellonella* larvae and mice [58]. The larvae were injected with both bacteria and phages (one hour later) into the last proleg. It was shown that the survival of the treated larvae after 72 h was dependent on the MOI of the administrated phages, i.e., 50%, 10%, and 0% for the MOI of 100, 10, and 1, respectively, for the Bϕ-R656 phage, and 60%, 27%, and 0% for MOI of 100, 10, and 1, respectively, for the Bϕ-R1836 phage. Each phage was specific to a particular strain of bacteria. Moreover, no pathological changes in tissues were observed in both phage-treated groups, whereas in C57BL/6 mice, acute pneumonia was induced by the intranasal administration of bacterial suspension. In the next stage, 4 h later, mice were treated with phages (at MOI = 10, MOI = 1, or MOI = 0.1) by the same route. The survival of the mice improved from 0% to 66% and 83% for Bϕ-R656 and Bϕ-R1836, respectively (for the highest MOI). Additionally, after the treatment with phages, the histological examination showed significantly less lung tissue damage, such as hemorrhaging in the alveolar space, compared to the untreated group. TNF-α and IL-6 levels in the phage-treated infected group were lower than in the bacteria-only treatment group, but still higher than in the negative control group.

It turns out that lysins from bacteriophages can be used in the treatment of bacterial infections [30,90,91]. Raz et al. described the effects of administering two bacteriophage-derived lysins to C57BL/6 mice with a skin infection or with pneumonia caused by *P. aeruginosa* [59]. After applying the bacterial cells to the specially prepared skin of mice, inflammation was induced, and then lysin PlyPa03 or PlyPa91 (in different doses: 200 µg or 300 µg) was applied topically. A dose-dependent reduction in bacterial load in the skin was obtained, which was more pronounced than when using lysin PlyPa91. To construct a mouse model of pneumonia, mice were intranasally administered a *P. aeruginosa* suspension twice. Thereafter, mice received two doses of lysin PlyPa91, two doses intranasally or intranasally and intratracheally (at 3 h intervals). An increase of survival rates was seen in both treatment groups, while the two-way route of lysin administration was much more effective (70% survival rate) in relation to the intranasal route (20% survival rate) after 10 days of observation. As noted, the route of delivery of the therapeutic agent is of great importance in the effectiveness of the therapy.

It has been established that various forms of phage preparations, including those taken by inhalation, for example, in the form of an aerosol [92] or a dry powder [93,94], have the potential to effectively control bacteria in pneumonia. Chang et al. reported the results of experiments that investigated the efficacy of dry powder phage therapy against *Pseudomonas aeruginosa* lung infection in neutropenic mice [60]. Inflammation was induced by passing bacteria into the trachea of mice. Then, 2 h later, the animals received an inhaled dose of the dry PEV20 phage preparation (2 × 10^7^ PFU/mg, MOI = 100). One day after the treatment, the histological examination of the mouse lung tissue revealed no harmful effects of the phage-only treatment group. In the mice infected with *Pseudomonas aeruginosa*, a significant reduction in inflammation-induced damage was observed. As noted for phage preparations, like for other therapeutic agents, new forms of administration to increase bioavailability and improve efficacy are desirable and may have significant benefits, and animal models appear to be a very useful tool in assessing these characteristics.

Nosocomial urinary tract infections caused by *Pseudomonas aeruginosa* have relatively high morbidity and mortality rates [95,96] and mainly affect catheterized patients [97]. There are reports in the literature of the mouse model (DBA1/LAC J mice) urinary tract infection caused by *P. aeruginosa* [46]. To induce acute inflammation, mice were injected with the bacteria transurethrally and then administered intraperitoneally with phages (5 × 10^10^ PFU/mL). Interestingly, treatment with phages resulted in enhanced intracellular killing of bacteria by murine splenic phagocytes, compared to the untreated group.

## 7. Animal Models of *Enterobacter* spp. Infections

Manohar et al. investigated the novel bacteriophage ELP140 against *Enterobacter cloacae* and assessed its therapeutic efficacy on infected *G. mellonella* larvae [83]. In this case, three doses of bacteriophages were required to achieve a complete survival rate of larvae after 96 h of observation. Similarly, triplicate bacteriophage administration was required to minimalize the bacterial load in larvae. As mentioned previously, a model of multiple bacterial infections was also used (with *E. cloacae*, *K. pneumoniae*, and *E. coli*), and after a single application of the phage cocktail, *E. cloacae* was completely eliminated.

Abbasifar et al. described an animal model of infection with *Cronobacter sakazakii* [51], which had been included in the *Enterobacter* genus before reclassification and known as *Enterobacter sakazakii* [98]. These studies used a model of *G. mellonella* infection where the bacterial suspension was administered into the hemolymph and which resulted in almost complete larval mortality without treatment [51]. To check the prophylactic effect of the GAP161 phage, the phage suspension was administered 1 h and half an hour before the injection of the bacteria, and as a result, much higher survival rates of the larvae 48 h after infection were observed. However, post-infection administration (1 h, 2 h, and 4 h after infection) of bacteriophages did not increase the survival rate. Interestingly, the effect of heat-inactivated bacteriophages on uninfected larvae was investigated, and no significant effect of high temperature (85 °C) on phages injected into the larvae was found.

**Figure 1:**
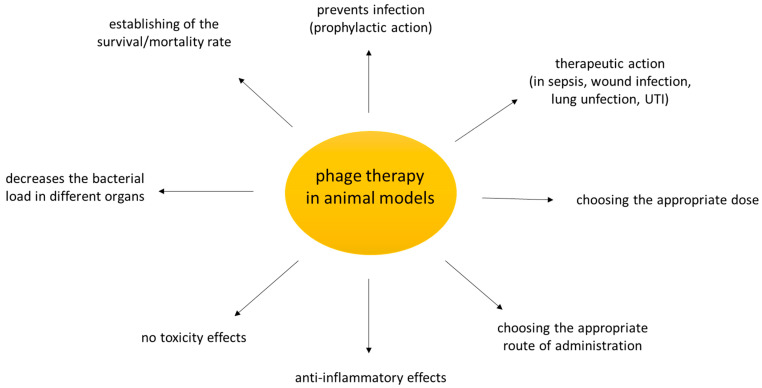
What could we learn from phage studies in animal models? Based on the research cited in the review.

Figure 1 shows the main achievements that can be learned from the use of animal models in preclinical studies of phage therapy.

Therefore, animal studies provide data of only limited value. Phage therapy is mostly applied in acute models of bacterial infections while clinical phage therapy usually deals with chronic cases. Furthermore, some routes of phage administration in animals will not be reproduced in patients (e.g., intraperitoneal route), so the data from such studies cannot be extrapolated to the human clinic.

## 8. Conclusions

Phage therapy is one of the most important tools offering a chance to successfully fight against multidrug-resistant bacteria. The ESKAPE group bacteria belong to extremely important alarm pathogens. Animal models of infection were discussed in this review and provided valuable information for phage therapy. Prophylactic effects of bacteriophages administered prior to infection have been repeatedly described using animal models. It is possible to obtain a lot of information on the pharmacokinetics of phages, as well as on their direct effect on the immune system, expressed in changes (or reduction) in the cytokine levels, as well as in the number or function of immunocompetent cells. It becomes possible to determine histology of the tissues of different organs and study the impact of administered phages on gut microbiota. The administration of phages at an appropriate dose after infection allows their therapeutic effect to be determined, as well as the most favorable route of phage delivery to be estimated. Despite the many differences between the organisms of various animals and the human body, all these achievements seem to be useful in the transfer of specific phage therapy to human medicine. The close contact between humans and pets creates a risk of zoonosis and interspecies transmission of pathogens [99]. There is a need for more studies in this area as many pathogens are zoonotic and emerge as a result of indiscriminate use of antibiotics in animal production.

## Figures and Tables

**Table 1:** Different routes of phage administration in selected animal models infected with bacteria from the ESKAPE group, their advantages and limitations.

Animal Model	Route of Phage Application	Advantages of this Route of Administration	Disadvantage of this Model in Phage Application
*G. mellonella*	into the last proleg (into the hemolymph) [51,52,53,54,58,82,83]	easy access of phages to bacteria	no possibility to choose an appropriate route of administration; no possibility to study different types of inflammation
*Drosophila melanogaster*	by injection [56]	easy access of phages to bacteria	difficult to apply (because of the size of the animal)
	with food (oral administration) [55]	simple way of administering	uncertainty that the correct dose will be taken; possibility of degradation in the digestive tract
Zebrafish with cystic fibrosis	into the yolk sac [84]	the place from which the embryo constantly takes nutrients—ensures that phages are delivered to the embryo	it does not correspond to the route of administration in humans
Mouse model of sepsis/bacteremia/peritonitis	intraperitoneal [55,61,62,63]	more direct (than i.m.) phage delivery to the site of infection	drugs are usually not administered by this route in humans
	intramuscular [55]	rapid distribution over the tissues due to the good vascularization of muscles; better (than i.p.) pharmacokinetics of phages	degree of adsorption depends on the blood supply to the specific muscle; more painful
Mouse model of pneumonia	intranasal [54,57,58,59]	easy way of applying phages	more difficult access of phages to the site of inflammation
	intratracheal [59]	easy access of phages to the site of inflammation	difficult way of application of phages
	as a dry powder inhalation [60]	easy and convenient way of administering	possible irritation of the respiratory tract
Mouse model of wound infection	topical [59,64]	direct application at the site of infection	limited range of the phages’ place of action
	intraperitoneal [64,85]	more systemic effects; more direct (than i.m. and s.c.) phage delivery to the site of infection	longer route of phages
	intramuscular [85]	rapid distribution over the tissues due to the good vascularization of muscles	degree of adsorption depends on the blood supply to the specific muscle; more painful
	subcutaneous [85]	-	small amount of preparation that can be administered
Mouse model of urinary tract infection	intravesical (no data available)	direct application to the site of infection	too much stress and pain
	intraperitoneal [46]	less stress to mice; more systemic effects	administration not directly to the site of infection
Mouse model of eye infection (keratitis)	topical [66,67]	direct application to the site of infection; easy access of phages to bacteria	-
